# “In the mood for ageing”: determinants of subjective well-being in older men and women of the population-based KORA-Age study

**DOI:** 10.1186/s12877-017-0513-5

**Published:** 2017-06-16

**Authors:** Karoline Lukaschek, Anushiya Vanajan, Hamimatunnisa Johar, Nina Weiland, Karl-Heinz Ladwig

**Affiliations:** 1 Institute of Epidemiology II, Helmholtz Zentrum München, German Research Center for Environmental Health, Ingolstädter Landstr. 1, 85764 Neuherberg, Germany; 2 Department of Psychosomatic Medicine and Psychotherapy, University of Giessen, Giessen, Germany; 3 Department of Psychosomatic Medicine and Psychotherapy, Klinikum rechts der Isar, Technische Universität München, Munich, Germany

**Keywords:** Subjective well-being, Mental health, Age-paradox, Population-based study

## Abstract

**Background:**

To investigate risk factors associated with low subjective well-being (SWB) in men and women (≥65 years) separately with a special focus on emotional distress.

**Methods:**

A cross-sectional analysis was conducted among 3602 participants (50.6% women) aged 65-90 years (mean age 72.8 years, SD ± 5.8) from the population-based KORA-Age study conducted in 2008/2009. SWB was assessed using the WHO-5 well-being index (score range: 0 to 100). SWB was dichotomized into “low” (score ≤ 50) and “high” (score > 50) SWB. The association between potential risk factors and SWB was assessed by logistic regressions analyses. Population-attributable risks (PARs) were calculated.

**Results:**

Low SWB was significantly higher in women than in men (23.8% versus 18.2%; *p* < 0.0001). The logistic regressions analyses revealed low income, physical inactivity, multimorbidity, depression, anxiety and sleeping problems to be associated with low SWB in both sexes. Living alone increased the odds of having low SWB in women, but not in men. Depression and anxiety were the strongest risk factors of low SWB among men (depression: OR: 4.19, 95% CI: 1.33-13.17, *p* < 0.05; anxiety: 8.45, 5.14-13.87, *p* < 0.0001) and women (depression: 6.83, 2.49-18.75 *p* < 0.05; anxiety: 7.31, 5.14-10.39, *p* < 0.0001). In both sexes, anxiety had the highest population-attributable risk (men: 27%, women: 41%).

**Conclusion:**

Our results call out for an increased focus on mental health interventions among older adults, especially for women living alone. Further research is needed to understand the paradoxical pattern of discrepant subjective well-being versus objective health in age.

## Background

The concept of subjective well-being (SWB) does not only refer to the absence of mental illness, but to a person’s positive evaluation of their psychological functioning and experience [1]. Three aspects of well-being can be distinguished: evaluative well-being (or life satisfaction), hedonic well-being (feelings of happiness, sadness, anger, stress, and pain), and eudaimonic well-being (sense of purpose and meaning in life) [2]. Thus, SWB is not a unitary construct: it is conceptualized as comprising an affective and cognitive component [3]. It is thought possible that the different aspects of SWB are affected differently, e.g. by age [2] or life-circumstances [4, 5]. Recent - yet debated [6] - findings from large population-based surveys identified a U-shaped relationship of well-being and age [2, 7] in western countries with well-being reaching its minimum around midlife. Complementary to these findings, a hill-shaped relationship over the life span has been shown for emotional distress (depression and anxiety), leading Blanchflower et Oswald [7] to the conclusion that the age of maximum mental distress is close to the age of minimum life satisfaction. Following this, middle aged individuals seem to be particularly vulnerable for low well-being and mental distress. Additionally, it is also expected that adverse life conditions such as mental and physical decline, disease and disability, as well as major adverse live events (e.g. the loss of power, independence and companionship) may accumulate with increasing age. Thus, as life expectancy increases, a particular challenge lies within understanding factors that affect the well-being of older adults in particularly.

A recent meta-analysis suggests that SWB is associated with a decreased risk in mortality in general [8]. Research on the determinants of SWB among older adults has identified social relationships [9], social capital [10], socioeconomic status [11] and psychosocial resources [12] as major factors. Most recently, Puvill et al. [13] showed that poor physical health was hardly related to lower life satisfaction in old age, whereas poor mental health was strongly related to lower life satisfaction.

However, with increasing age, sex differences in SWB grow stronger [14], with older women having lower levels of SWB than older men due to disadvantages in income, social relationships and socioeconomic status [14]. Contradictory evidence suggests that older men experience lower SWB than women as women prefer a diverse range of activities from which they gain happiness [15]. Additionally, older men may be more prone to difficulties in developing and continuing intimate relationships which could protect them from low SWB and negative mental health conditions [15].

Only few studies have applied the concept of mental distress to a broader, non-clinical understanding of subjective well-being [16]. Thus, we included in our analysis indicators of emotional distress (anxiety, depression and sleeping problems). To the best of our knowledge, no study to date has investigated sex differences in SWB with a special focus on emotional distress. Therefore, the present investigation aimed to examine the differences in SWB between men and women in an elderly community dwelling population (mean age: 73 years, SD ± 6) and the effect of depression and anxiety on well-being levels.

## Methods

### Study setting and study population

**Fig. 1:**
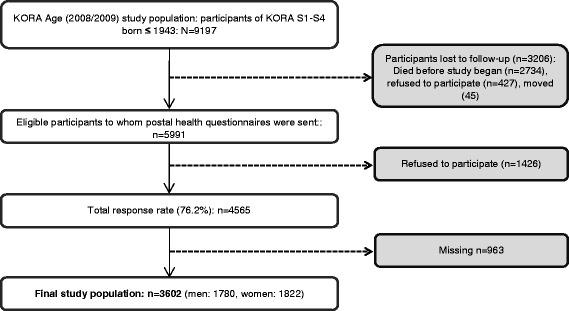
Flow chart of participants of the KORA-Age study and the final study population (*N* = 3602)

The KORA (Cooperative Health Research in the Region of Augsburg)-Age study is a population-based study conducted in southern Germany. Between 1984/1985 and 1999/2000, four cross-sectional population-based surveys were conducted in Augsburg and two surrounding counties with response rates from 79% in the first survey to 67% in the fourth survey [17]. KORA-Age includes all participants from those KORA surveys aged 65 years or older at the end of 2008, i.e. born in or before 1943. Details about KORA-Age have been reported elsewhere [18]. Briefly, 4127 people participated in a standardized telephone interview or postal health questionnaire. After excluding those with missing items in the selected variables, the current study analyzed a population of 3602 participants (men: *n* = 1750, 49.4%; women: *n* = 1822, 50.6%) (see Fig. 1 = flowchart).

The examinations and the interviews were performed by trained and experienced staff from the KORA study center in Augsburg. During the physical examination, standardised measurements of height, weight, waist circumference, blood pressure and serum lipids were performed as previously described [19]. BMI was calculated as weight in kilograms divided by the height in meters squared.

The KORA-Age study was approved by the Ethics Committee of the Bavarian Medical Association. Written informed consent has been obtained from the participants and all investigations have been conducted according to the principles expressed in the Declaration of Helsinki.

### Outcome: subjective well-being

Subjective well-being was assessed using the WHO-5 well-being index which was distributed through the postal health questionnaire. The WHO-5 is short scale for the measurement of positive subjective psychological well-being [20] and measures hedonic as well as eudaimonic aspects of well-being [21]. It consists of five positively phrased items which measure the participant’s well-being over the last 2 weeks *(felt cheerful and in good spirits / felt calm and relaxed /felt active and vigorous/woke up feeling fresh and rested/daily life filled with things that interest me)* [20]. The items are to be answered on a 6 point Likert scale ranging from 0 (not present) to 5 (constantly present) leading to a raw score range from 0 to 25. Raw scores are then transformed into a scale from 0 to 100 (representing the highest possible SWB score). According to Bech et al. [22] scores from 0 and 25 represent poor SWB, scores between 26 and 50 represent fair SWB, scores between 51and 75 represent good SWB and lastly, scores between 76 and 100 represent very good SWB. Additionally, the dichotomous cut off value for the WHO-5 SWB score was determined to be 50; all scores below 50 signify low SWB, whereas all scores above 50 signify high SWB [23]. Studies on the validity of the WHO-5 well-being index described high clinometric validity, and internal and external validity due to its high sensitivity and specificity [20].

### Socio-demographic and psychological variables

#### Socio-demographic variables

Low education was defined as less than 12 years of schooling. Income was dichotomized by classifying the variable ‘per capita income’ into less than 1000 euros per month (low income) and more than 1000 euros a month (high income). Living alone was assessed by asking the participants whether they lived alone or lived with a partner. *Lifestyle factors:* Smoking was classified into current regular or occasional smokers and non/ex-smokers. Leisure time physical inactivity versus activity was assessed by two interview questions. Each participant was asked: “How often do you carry out sports in the winter? How often do you carry out sports in the summer?” Sports were broadly considered in the context of elderly participant activities and included both bicycle riding and going on walks. Answers were given on a four-level graded scale (no activity, irregularly about 1 h/week, regularly 1 h/week, and regularly N2 h/week) [24]. A participant was classified as physically active if they regularly participated in sports during leisure time ≥ 1 h/week in either season. As standard procedure within the framework of the MONICA/KORA studies, physical activity was assessed as follows: participants were classified as ‘active’ during leisure time if they regularly participated in sports for at least 1 h per week; otherwise they were considered ‘inactive’. *Somatic variables:* Multimorbidity was defined as the co-occurrence of more than one disease conditions based on the Charlson Comorbidity Index [25]. *Psychological variables:* Depressive symptoms were measured by the 15-item German version of the Geriatric Depression Scale (GDS-15) where depression was depicted by a score of at least 10 [26]. Anxiety was assessed using the Generalized Anxiety Disorder scale [27]. Sleeping problems were evaluated by the Uppsala Sleep Inventory (USI) [28] compiling difficulties initiating and maintaining sleep as well as sleeping duration [29].

### Statistical analysis

Descriptive statistics were used to investigate the levels of subjective well-being with age. The Kruskal-Wallis Test was conducted to analyze sex differences in SWB. Analyses were stratified by sex (interaction term for sex and well-being both in categorical or continuous variable: *P* < .0001). Univariate associations between dichotomized covariates and SWB were analyzed through a chi-square test. Additionally, unadjusted associations of the two continuous covariates age and BMI with SWB were derived through the Mann-Whitney U test.

To further explore the association between exposure variables and low subjective well-being, four multinomial logistic regression models with different adjustments for covariates were performed. Model 1 was adjusted for socio-demographic variables (age, education, income and family status of living alone). Model 2 was adjusted for both socio-demographic variables and lifestyle factors (physical activity). It must be noted that at this stage the smoking variable was excluded as a lifestyle factor in the logistic regression models due to insignificant results from the chi-square tests on its association with SWB for both men and women. Model 3 included multimorbidity, whereas model 4 (full model) was additionally adjusted for psychological variables (anxiety, depression and sleep problems).

The c statistic was used to assess the model fit of the four multinomial logistic regression models.

Population-attributable risk (PAR) for each risk factor and the endpoint were calculated using the prevalence of the risk factor (prev) and the OR drawn from the logistic regression and applying the formula for computing adjusted PAR: PAR = ((prev * (OR −1)) / (prev * (OR -1) + 1)) * 100 [30].

Results were considered to be statistically significant when the *p* value was less than 0.05. The statistical software SAS Version 9.3 for Windows (SAS Institute Inc., Cary, NC, USA) was used to perform the aforementioned analysis.

The manuscript was prepared according to STROBE guidelines [31].

## Results

A total of 3602 participants were included in the analysis, among them 1780 (49.42%) were men. The age range for men was 65-89 years (mean age 72.8 years, SD ± 5.8), for women 65-90 years (mean age 72.8 years, SD ± 5.8).

### Subjective well-being among participants

**Fig. 2:**
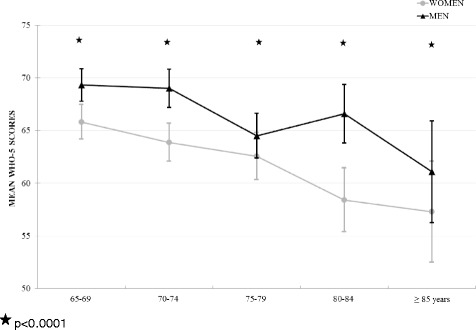
Association of mean SWB (WHO-5 score) levels with age categories

In total, 79% of the participants reported high levels of SWB (≥ cut-off value 50) compared to 21% experiencing low SWB. When stratified for sex, women reported significantly more often low levels of SWB than men (23.8% versus 18.2%; *p* < 0.0001). Fig. 2 depicts the mean SWB levels for men and women over 5-years age groups. With increasing age, a downward trend in SWB becomes evident in women presenting lower levels of SWB in all ages than men. However, the mean SWB scores remained far above the suggested cut-off for low SWB (SWB ≤ 50).

### Characteristics of participants

The mean age of men with low SWB was 73.8 years (SD ± 6.2 years) compared to a mean age of 72.6 years (SD ± 5.7) in men with high SWB. Similarly, the mean age of women with low SWB was 73.7 years (SD ± 6.0) compared to a mean age of 72.5 years (SD ± 5.7 years) in women with high SWB. These age differences were significant in men and women (*p* values 0.002 and <0.001). No significant differences in BMI between low and high SWB was observed in both sexes.

Further characteristics of men and women stratified for high and low SWB levels are presented in Table 1. Compared to men with high SWB, men with low SWB were significantly less educated, had lower income, were physically inactive and were experiencing multimorbidity, depression, anxiety and sleeping problems. There were no significant differences in SWB scores based on the male participants’ family status-living alone, BMI and smoking behavior. Similarly, women with low SWB had significantly lower income, lived alone, were physically inactive and experienced multimorbidity, depression, anxiety and sleeping problems. However, no significant associations were evident between low and high SWB and the female participants’ education level, BMI or smoking status.

**Table 1:** Association of socio-demographic variables, lifestyle factors, somatic variables and psychological variables with low and high SWB for men (*N* = 1780) and women (*N* = 1822)

Variable names	Low subjective well-being	High subjective well-being	*p*-value
Men (*N* = 1780)	*N* = 324 (18.20%)	*N* = 1456 (81.80%)	
Socio-demographic variables			
Low education	213 (65.74%)	857 (58.86%)	0.02
Low income	198 (61.11%)	689 (47.32%)	<.0001
Living alone	67 (20.68%)	268 (18.41%)	0.34
Lifestyle factors			
Smoking	25 (7.72%)	96 (6.59%)	0.48
Physical activity-inactive	153 (47.22%)	526 (36.13%)	0.0002
Somatic variables			
Multi-morbidity	305 (94.14%)	1221 (83.86%)	<.0001
Psychological variables			
Anxiety-yes	60 (18.52%)	29 (1.99%)	<.0001
Depression-yes	17 (5.25%)	5 (0.34%)	<.0001
Sleeping problems	183 (56.48%)	545 (37.43%)	<.0001
Women (*N* = 1822)	*N* = 434 (23.82%)	*N* = 1388 (76.18%)	
Socio-demographic variables			
Low education	374 (86.18%)	1177 (84.80%)	0.48
Low income	246 (56.68%)	684 (49.28%)	0.01
Family status-living alone	226 (52.07%)	626 (45.10%)	0.01
Lifestyle factors			
Smoking	26 (5.99%)	83 (5.98%)	0.99
Physical activity-inactive	267 (61.52%)	561 (40.42%)	<0.0001
Somatic variables			
Multi-morbidity	407 (93.78%)	1177 (84.80%)	<0.0001
Psychological variables			
Anxiety-yes	138 (31.80%)	66 (4.76%)	<0.0001
Depression-yes	40 (9.22%)	5 (0.36%)	<0.0001
Sleeping problems	295 (67.97%)	601 (43.30%)	<0.0001

### Association of socio-demographic, lifestyle, somatic and psychological variables with subjective well-being by logistic regression

Due to insignificant results in both sexes from unadjusted analyses, the smoking and BMI variables were not included in the logistic regression analyses. Table 2 describes the results for the four logistic regression models for men while Table 3 describes the findings for women.

**Table 2:** Association of socio-demographic variables, lifestyle factors, somatic variables and psychological variables with low subjective well-being for men (*N* = 1780)

Covariates	Model 1, OR (95% CI)	Model 2, OR (95% CI)	Model 3, OR (95% CI)	Model 4, OR (95% CI)
Age	1.03 (1.01-1.05)*	1.03 (1.01-1.05)*	1.02 (1.00 -1.04)	1.02 (1.00-1.04)
Low education	1.11 (0.85-1.44)	1.08 (0.83-1.41)	1.08 (0.83-1.41)	1.01 (0.76-1.34)
Low income	1.75 (1.34-2.29)**	1.71 (1.31-2.23)**	1.70 (1.30-2.22)*	1.65 (1.25-2.19)*
Living alone	1.31 (0.96-1.80)	1.26 (0.92-1.74)	1.28 (0.93-1.77)	1.19 (0.85-1.68)
Physical inactivity	-	1.40 (1.09-1.80)*	1.38 (1.07- 1.78)*	1.35 (1.03-1.76)*
Multi-morbidity	-	-	2.81 (1.72-4.58)**	2.66 (1.59-4.43)*
Anxiety-yes	-	-	-	8.45 (5.14-13.87)**
Depression-yes	-	-	-	4.19 (1.33-13.17)*
Sleeping problems	-	-	-	1.70 (1.31-2.21)**

Model 1 adjusted for socio-demographic variables (age, education, income and family status-living alone). Model 2 adjusted for socio-demographic variables and lifestyle factors (physical inactivity). Model 3 controlled for socio-demographic variables, lifestyle factors and somatic variables (multi-morbidity). Model 4 (full model) adjusted for socio-demographic variables, lifestyle factors, somatic variables and psychological variables (depression, anxiety and sleeping problems)

* *p* < 0.05, **p < 0.0001

**Table 3:** Association socio-demographic variables, lifestyle factors, somatic variables and psychological variables with low subjective well-being for women (*N* = 1822)

Covariates	Model 1, OR (95% CI)	Model 2, OR (95% CI)	Model 3, OR (95% CI)	Model 4, OR (95% CI)
Age	1.03 (1.01-1.05)*	1.01 (0.99-1.03)	1.01 (0.99-1.03)	1.00 (0.98-1.03)
Low education	1.01 (0.73-1.38)	0.98 (0.71-1.35)	0.97 (0.70-1-34)	0.93 (0.65-1.32)
Low income	1.48 (1.17-1.87)*	1.40 (1.11-1.77)*	1.39 (1.10-1.76)*	1.34 (1.03-1.73)*
Living alone	1.32 (1.04-1.68)*	1.34 (1.05-1.71)*	1.34 (1.05-1.71)*	1.43 (1.10-1.87)*
Physical inactivity	-	2.21 (1.76-2.77)**	2.18 (1.73-2.74)**	1.94 (1.51-2.50)**
Multi-morbidity	-	-	2.41 (1.58-3.69)**	2.48 (1.56-3.93)*
Anxiety-yes	-	-	-	7.31 (5.14-10.39)**
Depression-yes	-	-	-	6.83 (2.49-18.75)*
Sleeping problems	-	-	-	2.03 (1.59-2.61)**

Model 1 adjusted for socio-demographic variables (age, education, income and family status-living alone). Model 2 adjusted for socio-demographic variables and lifestyle factors (physical inactivity). Model 3 controlled for socio-demographic variables, lifestyle factors and somatic variables (multimorbidity). Model 4 (full model) adjusted for socio-demographic variables, lifestyle factors, somatic variables and psychological variables (depression, anxiety and sleeping problems)

*p < 0.05, **p < 0.0001

#### Results for men (Table 2)

Low income, physical inactivity and multi-morbidity were significantly associated with low SWB in all models. In the final model 4 (adjusted for socio-demographic variables, lifestyle factors, somatic variables and psychological variables such as depression, anxiety and sleeping problems) the associations of low income (OR: 1.65, 95% CI: 1.25-2.19), physical inactivity (1.35, 1.03-1.76) and multi-morbidity (2.66, 1.59-4.43) with low SWB were attenuated but still significant (*p* < 0.05). Additionally, we found that depression (4.19, 1.33-13.17, *p* < 0.05), anxiety (8.45, 5.14-13.87, *p* < 0.0001) and sleeping problems (1.70, 1.31-2.21, *p* < 0.0001) significantly increased the risk for low SWB.

#### Results for women (Table 3)

Low income, living alone, physical inactivity and multi-morbidity were significantly associated with low SWB in all models. In the final model 4 (adjusted for socio-demographic variables, lifestyle factors, somatic variables and psychological variables such as depression, anxiety and sleeping problems) the associations of low income (OR: 1.34, 95% CI: 1.03-1.73, *p* < 0.05) and physical inactivity (1.95, 1.51-2.50, *p* < 0.0001) with low SWB were attenuated but still significant. The associations of living alone (1.43, 1.10-1.87, *p* < 0.05) and multi-morbidity (2.48, 1.56-3.93, *p* < 0.05) with low SWB grew even stronger. Additionally, we found that depression (6.83, 2.49-18.75, *p* < 0.05), anxiety (7.31, 5.14-10.39, *p* < 0.0001) and sleeping problems (2.03, 1.59-2.61, *p* < 0.0001) significantly increased the risk for low SWB.

The model fit for the four models for both men and women was analyzed using the c statistic. The model fit was sufficient for all models. However, model 4 (for both men and women) had the highest c statistic (men: c = 0.71, women: c = 0.76).

### Population-attributable risk

For men, anxiety reached the highest value (PAR: 27.1%) followed by sleeping problems (22.3%), physical inactivity (11.7%), depression (3.8%) and living alone (3.5%). For women, the order was similar: anxiety (41.4%), sleeping problems (33.7%), physical inactivity (30.0%), living alone (16.7%) and depression (12.6%).

### Sensitivity analysis

We performed a linear regression using the continuous subjective well-being variable instead of the categorical variable in the full model (model 4 as described above). The results of the sensitivity analysis confirmed our pervious results (data not shown), except living alone which reached only borderline significance among women (*p* = 0.06) but was significant in men (*p* = 0.01).

## Discussion

### General findings

In this sample of 3602 community dwelling participants (age: ≥ 65 years) randomly drawn from the general population, well-being was generally high, with 79% of participants reporting high levels of SWB (≥ cut-off value 50). High levels of SWB in old age are consistent with previous findings from epidemiological studies [2, 9]. The stability of high levels of well-being and life-satisfactions despite of age-related physical decline has been coined “the age paradox” [13, 32], indicating that individuals possess enough resources to maintain well-being and positive attitudes toward life even when facing age-related risks for social losses and declining health. Albeit the general high levels of SWB, a slight decrease of well-being levels with age was observed in our study. Previous research has found a decline in well-being and life satisfaction around the age of 70 years [33].

The present analysis revealed that older women were at a higher risk of experiencing low SWB than older men. Our study adds to the surprisingly small body of literature that have found women to have lower levels of SWB due to disadvantages in health, partnership and material and financial resources [11, 14]. These results could be explained by the “social stratification theory” which affirms that members of the society who have been given more resources and opportunities would experience higher SWB [34]. Since women are more disadvantaged in terms of education, employment, and physical and mental health, older women would more likely face lower SWB than older men [14]. On the other hand, our results contradict theories such as the “role of preferences” (emphasizing that women have higher SWB due to their broad preferences in activities) or the “role of expectation” (arguing that women have higher SWB due to lower expectations) [15].

### What impacts well-being in old age?

It is generally assumed that age and sex, socioeconomic factors, somatic health and the quality of social relationships and social integration are important determinants of SWB [34]. In order to unravel the multifaceted dynamics of SWB, we performed stepwise regression analyses separately for men and women with an increasing number of factors included.

First, we assumed that increasing age would be a major determinant of low SWB [2] – however, the regression analysis confirmed that increasing age was only marginally associated with low SWB with a slightly stronger impact in men than in women. This encouraging finding suggests that increasing age is not inevitably associated with a decline in mood and quality of life [34–36]. Surprisingly, this holds true even for the very-old as recently shown by Wettstein et al. [16]: In their study population of 124 participants (mean age at baseline: 90.56 years, SD: ±2.92), a majority expressed remarkably high levels of eudaimonic and hedonic well-being with scores above the theoretical scale midpoints.

Following findings from Pinquart and Sorensen [11], we also expected low education and income to be associated with low SWB in later life. Our results confirm low socioeconomic status as a robust and independent risk factor for low SWB in both sexes, thereby underscoring the detrimental effect of severe economic restrictions in the elderly. Today more than ever, age-poverty represents a major societal and political issue. Given the complexity of the health status of the elderly and age-related pathologies [2, 37], it is no surprise that multi-morbidity and physical inactivity (compiling the spectrum of age related impairments and chronic disease conditions) remained as significant and robust predictors in the full model.

Living alone, loneliness, impaired social relationships and social disintegration are common age-related stereotypes in the general population which may substantially affect SWB [11]. In the present investigation, the impact of living alone on low SWB was significant only in women. Our results indicate that living alone has a dampening effect on the well-being of older women. Living with a partner in the same household is associated with more life satisfaction [36]. Women place greater value on social ties than men [38] and thus, living alone could make them particularly vulnerable to low SWB.

Surprisingly, the impact of emotional distress (anxiety, depression and sleeping problems) on the complex network of factors contributing to SWB has not received substantial attention so far. The major finding of our investigation was that anxiety and depression were by far the strongest contributor of low SWB in both elderly men and women in a fully adjusted model. Interestingly, the association of depression and well-being was substantially higher in women, thereby agreeing with preceding studies which revealed that older women are more vulnerable to low SWB due to depression [39, 40]. Our findings suggest that the age-related decrease in functional capacity as well as the increase in vulnerability and in health constraints limit the individual’s experience of life-satisfaction and well-being. Accordingly, de Beurs et al. [41] have shown in a population-based sample (*N* = 659) of older subjects (age range: 55-85 years) that anxiety was associated with increased disability and diminished well-being.

### Strengths and limitations

The strength of the study is the focus on elderly subjects taken from a well-established, large population-based data set and the use of the WHO-5 index, a widely accepted instrument to assess SWB. The study also benefits from the comprehensive focus on socio-demographic, lifestyle, somatic and psychological variables. Our analysis was based on cross-sectional survey and interview data. Thus, inferences about causality cannot be made. Generalization of our findings might be difficult due to culture-specific patterns of SWB and age perception as well as national income [42].

## Conclusion

In both sexes, anxiety and depression were the strongest risk factors for low SWB for both sexes, thereby emphasizing the importance of mental health on overall well-being. Our results call out for an increased focus on curative and preventative mental health interventions among older adults, especially for women living alone. Further research is needed to understand the somewhat paradoxical pattern of discrepant subjective well-being versus objective health in age.

## Abbreviations

OR: Odds ratio
PAR: Population-attributable risk
SD: Standard deviation
SWB: Subjective well-being
WHO: World Health Organization
